# Is amyotrophic lateral sclerosis/frontotemporal dementia an autophagy disease?

**DOI:** 10.1186/s13024-017-0232-6

**Published:** 2017-12-28

**Authors:** Zhiqiang Deng, Patricia Sheehan, Shi Chen, Zhenyu Yue

**Affiliations:** 1 Brain center, Zhongnan Hospital, Wuhan University, Wuhan, Hubei 430071 China; 2 Taihe Hospital, Hubei University of Medicine, Shiyan, Hubei 442000 China; 3 Department of Neurology, The Friedman Brain Institute, Icahn School of Medicine at Mount Sinai, New York, 10029 USA

**Keywords:** Amyotrophic lateral sclerosis, Frontotemporal dementia, Autophagy, Disease-associated genes, Autophagy-related genes

## Abstract

Amyotrophic lateral sclerosis (ALS) and frontotemporal dementia (FTD) are neurodegenerative disorders that share genetic risk factors and pathological hallmarks. Intriguingly, these shared factors result in a high rate of comorbidity of these diseases in patients. Intracellular protein aggregates are a common pathological hallmark of both diseases. Emerging evidence suggests that impaired RNA processing and disrupted protein homeostasis are two major pathogenic pathways for these diseases. Indeed, recent evidence from genetic and cellular studies of the etiology and pathogenesis of ALS-FTD has suggested that defects in autophagy may underlie various aspects of these diseases. In this review, we discuss the link between genetic mutations, autophagy dysfunction, and the pathogenesis of ALS-FTD. Although dysfunction in a variety of cellular pathways can lead to these diseases, we provide evidence that ALS-FTD is, in many cases, an autophagy disease.

## Background

Though various cellular defects are noted in Amyotrophic Lateral Sclerosis (ALS) and Frontotemporal dementia (FTD) including dysregulation of RNA processing, protein aggregation, and oxidative stress, the detailed disease mechanisms remain poorly understood [1, 2]. Emerging evidence from genetic cases points to a role of autophagy in ALS-FTD. Here, we review how specific disease-linked mutations affect proper autophagic function and protein degradation leading to the speculation that ALS-FTD is, at least in part, an autophagy disease.

### ALS and FTD

ALS is an adult-onset devastating neurodegenerative disorder characterized by progressive degeneration and deterioration of upper and lower motor neurons of the primary motor cortex, spinal cord and brain stem [3]. FTD is a form of dementia characterized by focal atrophy of the frontal and anterior temporal lobes of the brain, called frontaltemporal lobar degeneration (FTLD) [4]. The comorbidity of these diseases in patients and the shared genetic risk factors suggest ALS and FTD represent a continuum of neurodegenerative disorders [5]. A common pathological hallmark of both ALS and FTD is the presence of cytoplasmic protein aggregates and inclusions in affected neurons and glia cells [6–8], suggesting that an impairment in protein degradation may contribute to the disease process. In eukaryotic cells, the clearance of toxic-aggregated proteins is critical for cell survival, which relies mainly on two protein degradation systems: the ubiquitin-proteasome system (UPS) and autophagy [9, 10]. The UPS is mainly used for the degradation of short-lived proteins, while autophagy is preferentially used for the selective degradation of long-lived proteins and damaged organelles [11]. While the dysfunction of either the UPS or autophagy has been implicated in the formation of ALS-FTD-linked protein aggregates, accumulating evidence suggests that proper functioning of autophagy is the major determinant of motor neuron survival in ALS [12–15].

### Autophagic processing

Autophagy is a highly conserved catabolic cellular pathway used to degrade proteins and organelles at a basal level as well as pathogens and protein aggregates under pathological conditions. The endpoint of all autophagic pathways is the lysosome though, the three major autophagic pathways (i.e., microautophagy, chaperone-mediated autophagy, and macroautophagy) utilize different signaling molecules and protein machinery to prompt lysosomal degradation. This review will focus on the role of macroautophagy (hereafter referred to as autophagy) in ALS-FTD.

Canonical autophagy is under the control of mechanistic target of rapamycin complex 1 (mTORC1), a master regulator of diverse signaling pathways, which is regulated by cellular nutrient levels. Under nutrient-rich conditions, mTORC1 is active, suppressing the induction of autophagy by phosphorylating Unc51-like kinase 1 (ULK1) and Transcription Factor EB (TFEB). Starvation conditions inhibit the activity of mTORC1 and allow the induction of autophagy through the dephosphorylation of ULK1 and TFEB. Upon dephosphorylation, TFEB translocates to the nucleus where it functions to upregulate autophagy and lysosome-associated genes thus promoting autophagic degradation [16]. Once ULK1 is dephosphorylated, it forms a complex that can then phosphorylate Beclin1 [17] and Atg14L [18], two protein subunits in the class III phosphoinositol-3-phosphate kinase VPS34 complex. ULK1-mediated Atg14L phosphorylation activates VPS34 kinase activity, which is required for the nucleation of the phagophore membrane. The phagophore then expands to form the isolation membrane and engulf cytoplasmic contents. This elongation of the isolation membrane is dependent on two ubiquitin-like conjugation systems. The first involves the covalent conjugation of Atg12 to Atg5, a process carried out by Atg7 and Atg10. Atg5-Atg12 complex then binds Atg16. The second requires Atg7 and Atg3, assisted by Atg5-Atg12-Atg16, leading to the conjugation of phosphoethanolamine to LC3 [19]. This lipidation of LC3 allows it to associate with the autophagosome membrane and aid in cargo sequestration by associating with various autophagy receptors. Eventually, the membrane closes and forms a double membrane structure referred to as an autophagosome [19]. Following cargo sequestration, autophagosomes are trafficked along microtubule tracks. During this transport, autophagosomes can then fuse with compartments of the endocytic pathway to form an intermediate structure called an amphisome, which will then fuse with lysosomes for cargo degradation [20]. Additional studies investigating the cellular machinery for these fusion events have identified endosomal or lysosomal proteins (i.e., Rab7, LAMP1, HOPS complex proteins) and various SNARE proteins (i.e., Syntaxin17, SNAP29, VAMP7/8) as key mediators of autophagosome-lysosome fusion [21].

Autophagy was initially characterized as a non-selective degradation process. However, accumulating evidence shows that cargo such as damaged mitochondria and aggregated proteins can be selectively degraded by autophagy to maintain intracellular homeostasis [22–24]. This process of selective autophagy is carried out by autophagy receptors, which recognize specific cargo (including disease-related proteins) through protein modifications of receptors in response to proteotoxic stress [25].

Although growing evidence points to an association of autophagic function and the development of ALS and FTD [8, 26], the precise role of autophagy in the pathogenesis of these diseases remains elusive. Here, we review recent evidence for the role of ALS-FTD-associated genes in autophagic processing.

### ALS-FTD-associated genes and autophagy

#### C9ORF72

The GGGGCC (G_4_C_2_) hexanucleotide repeat expansion in C9ORF72 is the most common genetic cause of ALS-FTD [27]. Multiple pathogenic mechanisms are thought to underlie the pathology associated with the G_4_C_2_ expansion. This alteration of C9ORF72 may lead to epigenetic changes [28] that result in decreased mRNA levels and the loss-of-function of C9ORF72 [27]. Alternatively, G_4_C_2_ expansion containing transcripts may be subject to repeat-associated non-ATG (RAN) translation that could result in a toxic gain-of-function of dipeptide-repeat proteins [29–31]. Multiple groups have initiated studies to develop therapeutic interventions aimed at reducing the toxicity of dipeptide-repeat proteins using gene depletion, peptide, or small molecule strategies to decrease RAN translation [32]. Pathologically, post-mortem analysis of patients carrying the G_4_C_2_ expansion in C9ORF72 reveals the presence of RNA foci and ubiquitin/p62 positive inclusions [33–35]. Given these findings, much interest has arisen in understanding the potential link between C9ORF72 and autophagic regulation. Accumulating evidence demonstrates the relevance of C9ORF72 to autophagy, though its precise role in regulating autophagy is still unknown. Evidence suggests C9ORF72 acts in a multi-protein complex with SMCR8 and WDR41 to regulate the expression and activity of ULK1 [36]. Further, knockdown of C9ORF72 in MEF cells and mice impairs ULK1-mediated autophagy nduction, suggesting that C9ORF72 promotes autophagy [36, 37]. Acting in the same complex, C9ORF72 functions as a guanine nucleotide exchange factor (GEF) to activate Rab8a and Rab39b, two Rab GTPases with known functions in autophagy [38]. In the same study, depletion of C9ORF72 partly impaired autophagy and enhanced aggregation of TDP-43 and accumulation of p62 aggregates in neurons [38]. Additional studies indicate that C9ORF72 controls the Rab1a-dependent recruitment of the ULK1 complex to the phagophore in order to regulate the initiation of autophagy [39]. Again, the reduction of C9ORF72 protein levels was shown to attenuate autophagy and enhance the formation of p62-positive puncta in cells and primary neurons [39]. Together, these results indicate that C9ORF72 acts as a positive regulator of autophagy with its depletion leading to autophagic dysfunction and protein aggregation. However, in addition to regulating autophagy through direct association with the ULK1 autophagy initiation complex, C9ORF72 is indirectly involved in autophagy through regulation of mTORC1. Active mTORC1 inhibits autophagy by negatively regulating ULK1 activity and TFEB-mediated transcription of autophagy genes [40–42]. In the context of ALS-FTD, depletion of C9ORF72 was shown to impair mTORC1 activity by decreasing the response of mTORC1 to amino acid availability [43], reducing the phosphorylation of S6 K, a substrate of mTORC1, and enhancing autophagic flux [44]. In contrast to the findings discussed earlier, these results suggest that depletion of C9ORF72 enhances autophagy through negative regulation of mTORC1. As it is still unclear what the precise role of C9ORF72 is in autophagy, further study is required to determine whether C9ORF72 depletion results in enhanced or impaired autophagy regulation.

#### TBK1

TANK-binding kinase 1 (TBK1) was recently identified as an ALS-FTD causal gene [45, 46]. ALS-FTD-linked TBK1 mutations are proposed to be loss-of-function mutations as patients show decreased mRNA and protein expression [45, 46]. TBK1 has been implicated in various autophagy-related pathways including antibacterial autophagy (xenophagy), mitophagy, and macroautophagy [47–51]. TBK1 phosphorylates autophagy receptors including p62 and OPTN enhancing the elimination of intracellular pathogens such as Mycobacteria and Salmonella, as well as damaged mitochondria [48–51]. Recent studies have shown that ALS-related mutations of TBK1 inhibit mitophagy by blocking autophagosome formation, disrupting the OPTN-TBK1 interaction [46, 52, 53], and failing to translocate to damaged mitochondria [54]. TBK1 is necessary for the maturation of the autophagosome, which is required for the degradation of p62 and its affiliated cargo [50]. Overexpression of wild-type TBK1, but not the kinase dead variant, facilitates clearance of mutant SOD1, another ALS associated protein [55, 56]. These results indicate that ALS-FTD-associated mutations of TBK1 disrupt autophagy through loss of TBK1-regulated clearance of autophagic cargo, including damaged mitochondria and disease-prone proteins.

Recently, a link between TBK1 and C9ORF72 has been reported. TBK1 phosphorylates SMCR8 at S402 and S796, which forms a complex with C9ORF72 and WDR41 to control autophagy flux [38]. Depletion of SMCR8 impairs autophagy, which can be rescued by expression of phosphomimetic variants of SMCR8 but not non-phosphorylatable variants of SMCR8, suggesting a critical role of TBK1 kinase activity in regulating autophagy through SMCR8. Moreover, knockdown of TBK1 enhances the accumulation of p62 puncta in neuronal cells, which can be rescued by phosphomimetic SMCR8 but not non-phosphorylatable SMCR8. These results imply that TBK1 and C9ORF72 may share a common pathway in regulation of autophagy through SMCR8 [38]. Together, ALS-FTD-causal mutations in TBK1 appear to cause the dysregulation of multiple aspects of autophagic function including mitophagy, toxic protein clearance, and C9ORF72/SMCR8 cellular pathways.

### Optineurin (OPTN)

OPTN is involved in a variety of cellular functions, including signal transduction, vesicle trafficking, and axon homeostasis [57]. OPTN is a known autophagy receptor, which promotes the clearance of protein aggregates, infected pathogens, and mitochondria [48, 49, 55]. Several studies report that genetic mutations in OPTN enhance the risk of ALS [58–63]. Multiple lines of evidence suggest that ALS related OPTN mutations impair autophagy. For example, ALS patients with OPTN mutation (E478G) show TDP-43 positive neuronal intracytoplasmic inclusions in medullary and spinal motor neurons [58, 59]. In addition, Wild et al. have shown that ALS-linked OPTN mutations (E478G and F178A) are unable to rescue antibacterial autophagy to the level of wild-type OPTN [48]. More recent studies have shown that ALS-linked mutations of OPTN (Q398X, E478G) disrupt its association with myosin VI, an association that is required for autophagosome maturation. This disruption leads to disturbances in the activation of ER stress, dysfunction in protein secretion, Golgi fragmentation, and autophagosome-lysosome fusion [64].

Post-translation modifications, including phosphorylation and ubiquitination, of OPTN enhance its function in autophagy. OPTN can be phosphorylated at various serine residues by TBK1. In antibacterial autophagy, TBK1 phosphorylates OPTN at S177, which enhances its binding affinity with LC3 and promotes autophagic clearance of cytosolic Salmonella [48]. While in mitophagy, TBK1 phosphorylates OPTN at S473 in the UBA domain, which enhances its binding affinity to ubiquitin chains [49]. In addition, phosphorylation of OPTN at Ser473, which is close to ALS-linked mutant site E478G, enhances its binding to ubiquitin chains and its clearance of damaged mitochondria [49]. However, interactome analysis in another study shows this mutation reduces OPTN interactions with binding partners, including proteins which functions in ubiquitin-dependent protein degradation and ER transport [65]. OPTN can also be ubiquitinated at multiple lysine residues by the tumor-suppressor HACE1, which promotes its interaction with p62 to form an autophagy receptor complex and accelerate autophagic flux [66]. Recently, Shen et al. have shown that compared with wild type OPTN, ubiquitin-binding domain mutants including the ALS-linked E478G and D474N mutations, are defective in clearing inclusion bodies formed by truncated TDP-43 [67]. The same group has also shown that the mutants act as dominant-negative traps to compromise the maturation of autophagosomes, which leads to defects in OPTN-mediated selective autophagy [67]. Future experiments are required to determine whether the phosphorylation or ubiquitination of OPTN is impacted in ALS linked mutations.

As discussed above here, post-translational modifications play a crucial role in modifying the function of OPTN in autophagy. Either phosphorylation or ubiquitination of OPTN increases autophagy and lead to the clearance of autophagy substrates. Therefore, it is conceivable that enhancing phosphorylation or ubiquitination of OPTN could be a novel therapeutic target for ALS patients carrying OPTN mutations.

### p62 (SQSTM1)

p62 is an autophagy receptor that plays a major role in the clearance of protein aggregates [68]. Moreover, p62 itself is degraded by autophagy and thus can be used as a general marker for autophagic flux [68, 69]. p62 functions in diverse signaling pathways including proteasomal signaling, amino acid sensing, DNA damage response, and oxidative stress [63, 70, 71]. Pathological analyses of postmortem brains reveal that p62 associates with various disease-relevant protein aggregates [72–74]. Due to the numerous roles of p62 in the clearance of pathogens, protein aggregates, and various client proteins, it has become the most well-characterized autophagy receptor [68, 69]. The UBA domain of p62 can be phosphorylated by Casein kinase 2 (CK2), TBK1, and ULK1. These phosphorylation events increase the affinity of p62 for ubiquitinated cargo and enhance the clearance of ubiquitinated proteins in selective autophagy [50, 68, 75]. Additionally, p62 can be ubiquitinated by TRIM21 and Keap1 to regulate the sequestration required for clearance of ubiquitinated proteins in selective autophagy [76, 77]. More recently, it has been reported that upon ubiquitin stress (e.g., proteasome inhibition), p62 undergoes ubiquitination which disrupts the dimerization at its UBA domain allowing p62 to recognize and bind polyubiquitinated cargoes [78].

Mutations in p62 have also been found in ALS-FTD patients [63, 79–81]. A recent report indicates that some rare variants of p62 in the UBA domain increase FTLD risk [80]. While various mutations in p62 have been identified in ALS-FTD, the relevance of these mutations to disease risk is currently unknown [82]. Limited evidence suggests that disease-associated mutations may confer loss-of-function of p62 in selective autophagy [83, 84]. Knockdown of p62 in zebrafish causes a locomotor phenotype that is improved under mTOR inhibition or re-expression of wild-type p62, but not ALS-FTD-associated UBA domain mutant (P392L) p62 [83]. Further, the ALS-FTD-associated p62 LIR mutant, L341 V, is defective in recognition of LC3B [84]. This recognition is required for p62-mediated delivery of ubiquitinated cargo to the autophagosome suggesting this ALS-FTD related mutation results in failed selective autophagy. Finally, recent evidence suggests that p62 regulates the levels and/or functions of other ALS-FTD disease proteins (e.g., SOD1 and TDP-43) [85, 86], which is consistent with a prominent role of the misregulation of p62 in the disruption of protein homeostasis in ALS-FTD.

### Ubiquilin 2 (Ubqln2)

Ubqln2 is ubiquitin-like receptor protein that shuttles polyubiquitinated cargo to the proteasome for degradation via its UBA domain [87]. While the role of Ubqln2 in the UPS has been well established, emerging evidence of an interaction between Ubqln2 and LC3 suggests this protein also has a role in autophagy [88, 89]. Multiple mutations in the PXX repeat region are linked to ALS-FTD [63, 90, 91]. In transgenic rats, neuronal expression of a disease-linked Ubqln2 mutation (P497H) induces the formation of protein aggregates positive for p62 and LC3, however, deletion of Ubqln2 has no effect [92]. As the effects of this mutation do not mimic loss-of-function, this finding suggests that the ALS-FTD-linked Ubqln2 mutation (P497H) confers a gain of toxic function [92]. Furthermore, expression of Ubqln2 mutants results in the accumulation of polyubiquitinated proteins (e.g., TDP-43) in a UBA domain-dependent manner [93]. However, a more recent study has shown that Ubqln2 clears aggregates via the proteasome by interacting with the chaperone protein HSP70 [94]. Disease mutations in Ubqln2 are defective in HSP70 binding leading to the accumulation of aggregated proteins, which suggests loss-of-function mutations in Ubqln2 may underlie ALS-FTD [94].

### TDP-43

TDP-43 is an RNA- and DNA-binding protein, which harbors RNA recognition motifs (RRM) and a C-terminal glycine-rich domain (GRD) that are required for its functions in nucleic acid binding and protein-protein interactions, respectively [95]. TDP-43 was detected as a major component of the ubiquitin-positive inclusions in ALS-FTD [96]. During the past several years, the identification of different mutations in ALS-FTD has shown that TDP-43 is a causative gene to these diseases [97]. It is reported that TDP-43, especially aggregated TDP-43, is cleared by autophagy [85, 98, 99] and autophagy activators reduced the formation of TDP-43 positive inclusion in TDP-43 overexpression mouse models [100]. High levels of truncated TDP-43, called TDP-25, are a prominent feature of ALS-FTD and are associated with reduced induction of autophagy [101]. Others have reported that depletion of TDP-43 reduces the mRNA level of Atg7, by destabilizing Atg7 mRNA, which causes an impairment in autophagy and the accumulation of polyubiquitinated proteins and p62 in neuroblastoma cells [102]. However, a recent report showed that loss of TDP-43 increased the biogenesis of autophagosomes and lysosomes by inhibiting mTORC1 activity and enhancing TFEB activity while also disrupting the fusion of autophagosomes and lysosomes [103]. Although these studies show seemingly opposite effects of the loss of TDP-43 on autophagy induction, they each suggest an essential role of full-length TDP-43 in proper autophagy regulation and indicate the dysfunction of multiple steps of autophagy is linked to TDP-43-mediated ALS-FTD.

Stress granules are aggregations of RNA and RNA-binding proteins that are dynamically assembled and disassembled when cells are insulted by stress [104]. These stress granules are essential to the maintenance of RNA homeostasis, the dysregulation of which is highly implicated in ALS pathogenesis [104, 105]. TDP-43 is a major component of stress granules in ALS while another ALS-related protein, FUS, is found in these granules in a subset of cases [106, 107]. FUS is also an RNA binding protein and mutations in FUS account for around 5% of familial ALS cases and result in the loss-of-function of FUS [108, 109]. ALS-associated FUS mutation (R521C) accumulates FUS positive stress granules upon stress conditions and disrupts the release of FUS from stress granules in cultured neurons [110]. Moreover, ALS-linked mutant FUS affects the assembly and morphology of stress granules [111]. As stress granules are degraded by autophagy, it is conceivable that mutant FUS decreases the dynamics of stress granules and leads to their accumulation by disrupting autophagic clearance of these structures [105, 112]. One study suggested that prolonged stress granule formation might contribute to the pathogenesis of ALS. Interestingly, treatment of small molecule inhibitors of eIFα, which is directly correlated with stress granule formation, caused reduction of TDP-43 toxicity [113]. Decrease of TDP-43 toxicity by the same inhibitors has been also shown in other model systems [114], providing the support to the idea of targeting TDP-43 and stress granule in therapeutic development.

### VCP

Valosin-contain protein (VCP), also called AAA-ATPase p97, functions in diverse signaling pathways, including endoplasmic reticulum (ER)-associated degradation, transcriptional regulation, DNA damage, and membrane dynamics [115–118]. Loss-of-function mutations in VCP are linked to several human degenerative diseases, including inclusion body myopathy (IBM), ALS-FTD [119, 120]. Histopathological analysis of VCP-associated neurodegenerative diseases show that affected tissues contain prominent inclusions containing ubiquitin and TDP-43 [121–123], and an accumulation of LC3 and p62 [124]. These findings, along with results from recent publications, suggest that VCP is involved in autophagy and specifically functions in the delivery of cargo to lysosomes for degradation [125]. Evidence from yeast has shown an essential role of the VCP orthologue, Cdc48, in the formation of autophagosomes [126] and multiple groups have reported the loss of VCP expression or activity leads to an accumulation of autophagosomes which fail to degrade aggregated proteins due to impaired autophagosome-lysosome fusion [104, 106]. Additional studies have found that mTORC is disrupted in VCP-related diseases, which leads to increased autophagosome biogenesis [127]. This finding, along with recent studies showing a critical role of VCP in the elimination of damaged lysosomes by autophagy, suggests the loss of VCP results in the overproduction of autophagosomes, a disruption in autolysosome formation, and the dysfunction of lysosomes [128]. Other studies have also shown a role for VCP in mitophagy and stress granule degradation [129], suggesting that VCP mutations result in ALS-FTD by disrupting multiple autophagic pathways.

### SOD1

The Cu/Zn superoxide dismutase (SOD1) is an ALS causative gene in which mutations account for about 1% of sporadic and 10% of familial cases [130]. Mice expressing mutant SOD1 show an accumulation of aggregated proteins (including mutant SOD1) in the spinal cord, which is comparable with findings in human ALS [131]. Accumulating evidence demonstrates that genetic mutations of SOD1 induce ALS through a dominant toxic gain-of-function rather than the loss of enzymatic function [132, 133]. Previous reports have shown that autophagy can degrade mutant SOD1, preventing its toxicity [134] and antagonizing its cytoplasmic accumulation [135]. Recently, p62 and another ALS-associated gene, ALS2, have been shown to have additive protective roles in antagonizing the toxicity induced by mutant SOD1 [136]. In addition, it has been reported that inhibition of motor neuron autophagy in SOD1-G93A mice induces neuromuscular denervation in the early stages of the disease [137]. Another recent article has shown that bosutinib, which boosts autophagy, can improve the survival of ALS iPSC-derived motor neurons from patients with familial ALS caused by mutations in SOD1 [138]. It is likely that the autophagy-lysosome pathway is disrupted at asymptomatic stages in familial ALS-associated human SOD1-G93A as suggested in a transgenic model [139]. Additionally, the expression of TFEB and Beclin 1, two autophagy-related proteins, is altered in NSC-34 cells and in transgenic mice expressing SOD1-G93A mutation [140, 141]. In other studies, hyperactivity of the autophagy/lysosome pathway was detected in motor neurons of SOD1-linked ALS mice [142], which may account for the accumulation of autophagosomes in transgenic mice expressing mutant SOD1-G93A [143]. Taken together, these studies suggest that mutant SOD1 leads to a misregulation in autophagy. It is intriguing to propose that the disturbance of autophagy-lysosome pathway contributes to the toxicity induced by mutant SOD1, and modulation of autophagy may be a potential strategy for SOD1-mediated ALS treatment.

### ALS2 (Alsin)

Mutations in ALS2 were identified in juvenile cases of motor neuron diseases, including ALS [144–146]. Previous reports showed that ALS2 activates Rab5 to regulate the trafficking, fusion, and maturation of endosomes [147–149]. The pathogenic mutations in ALS2 fail to activate Rab5 and disrupt the formation of amphisomes, a hybrid organelle formed through the fusion of autophagosomes and endosomes [150]. Loss of ALS2 in SOD1-H46H transgenic mice results in the accumulation of insoluble high molecular weight SOD1 but also ubiquitinated proteins and p62, while overexpression of ALS2 reduces the toxicity of mutant SOD1 in cultured motor neuronal cells [151]. More recently, ALS2, together with p62, was shown to play an additive protective role in antagonizing the toxicity of mutant SOD1 by promoting the clearance of insoluble mutant SOD1 [136]. These results suggest that the loss-of-function of ALS2, associated with impaired autophagy-induced protein clearance, leads to motor neuron disease associated pathogenesis.

### VAPB

Vesicle-associated membrane protein-associated protein B (VAPB, also known as ALS8) is a member of highly conserved VAP family of ER transmembrane proteins [152]. VAPB plays a role in diverse functions including regulating calcium homeostasis in mitochondria and membrane trafficking in dendrites [153, 154]. More recently, Gomez-Suaga et al. have shown that VAPB and PTPIP51, a mitochondrial associated protein, regulate autophagy by modulating calcium delivery at ER-mitochondria contact sites [155]. Following the initial identification of a dominant missense mutation of VAPB in patients with a slowly progressive form of ALS [156], other mutations of VAPB have also been found to cause ALS [157, 158]. Knock-in mice expressing mutant VAPB-P56S show accumulation of cytoplasmic inclusion containing mutant VAPB and ubiquitinated proteins in motor neurons selectively, implicating that defects in autophagy contribute to the pathogenesis of VAPB-mediated ALS [159].

### SigR1

Sigma receptor-1 (SigR1, also known as ALS16) is an ER chaperone protein involved in calcium signaling, ion channel activity, synaptic plasticity, and neuronal survival [160]. A mutation in SigR1 (E102Q) was identified as causative of juvenile ALS-FTD [161]. Emerging evidence has shown the association of SigR1 with autophagy. Vollrath et al. reported that knockdown of SigR1 in HEK 293 or NSC34 cells leads to impaired autophagic degradation. Additionally, in cells stably expressing autophagy reporter RFP-GFP-LC3, depletion of SigR1 disrupts the fusion of autophagosome to lysosome [162]. More recently, the SigR1-E102Q protein was found to form aggregates in ER, which alters the structure of the ER and further results in autophagy impairment by affecting the fusion of autophagosomes to lysosomes. Moreover, similar results were found in primary lymphoblastoid cells derived from familial ALS patients harboring the SigR1-E102Q mutation [163]. These studies imply that mutations in SigR1 result in the loss of its function in maintaining ER morphology and in autophagic processing.

## Conclusions

**Fig. 1:**
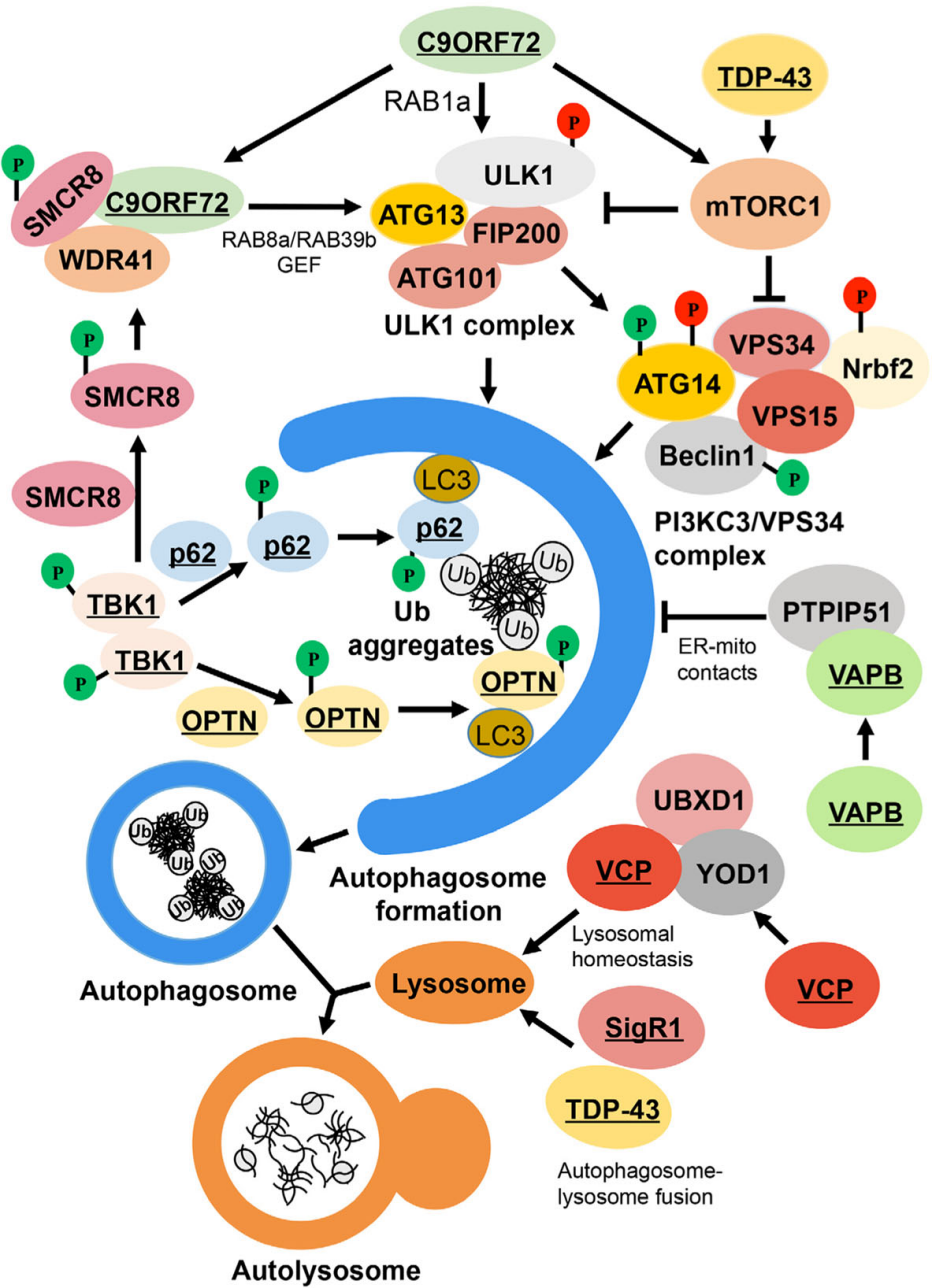
Interactions of ALS/FTD associated genes with autophagy pathway. Several ALS/FTD genes take part in the selective autophagy pathway, which controls the clearance of ubiquitinated proteins and damaged organelles. Autophagy kinase TBK1 phosphorylates two known autophagy receptors p62 and OPTN to facilitate the clearance of ubiquitinated proteins. TBK1 also phosphorylates SMCR8 to regulate the formation of SMCR8, WDR41 and C9ORF72 complex, which acts as a GEF (GDP/GTP exchange factor) for RAB8a/13b, which is associated with the dynamics of autophagosomes. The C9ORF72 complex potentially regulates the ULK1 kinase activity. C9ORF72 can interact with Rab1a and ULK1 complex to regulate autophagy initiation. Moreover, C9ORF72 can negatively regulate autophagy by increasing mTORC1 activity. TDP-43 regulates mTORC1 activity; however, it can enhance autophagosome–lysosome fusion in an mTORC1 independent manner. VAPB, together with PTPIP51, deregulates autophagy flux by controlling the ER-mitochondria contacts. VCP, which cooperates with UBXD1 and YOD1, maintains lysosomal homeostasis. Underlined genes are linked to ALS/FTD. Abbreviations: ALS: Amyotrophic lateral sclerosis; FTD: Frontal-temporal dementia; TDP-43: Transactive response DNA-binding protein 43; SMCR8; Smith-Magenis Chromosome Region gene 8; WDR41: WD repeat domain 41; ULK1: Unc-51 Like Autophagy Activating Kinase 1; C9ORF72: Chromosome 9 open reading frame 72; GEF: Guanine nucleotide exchange factor; mTORC1: mechanistic target of rapamycin complex 1; TBK1: TANK-binding kinase 1; OPTN: Optineurin; LC3: microtubule-associated protein1 light chain 3; VAPB: Vesicle associated membrane protein associated protein B; VCP: Valosin containing protein; SigR1: Sigma receptor-1; UBXD1: ubiquitin regulatory X (UBX) domain-containing protein 1; YOD1: yeast OTU deubiquinating enzyme 1; ER: endoplasmic reticulum; mito: mitochondria; PTPIP51: protein tyrosine phosphatase interacting protein 51.

While dysfunction in multiple cellular processes can underlie the development of ALS/FTD, an assortment of evidence demonstrates a role of autophagy dysfunction in these diseases. The above evidence suggests that different ALS/FTD linked mutations lead to dysfunction in all aspects of autophagy ranging from initiation and cargo recognition to autophagosome fusion with endosomes and lysosomes (Table 1 and Fig. 1). Dysfunction in any of these steps of autophagy leads to a disruption in protein homeostasis and results in the accumulation of toxic protein aggregates. While general enhancement of autophagy is likely to reduce these protein aggregates, the chronic upregulation of autophagy biogenesis can lead to an imbalance of autophagosome formation and clearance, resulting in neurotoxicity. More directed therapeutic interventions targeted at specific regulation of autophagy receptors or proteins are necessary to combat toxic protein accumulation effectively. For instance, specific compounds (e.g., HSF1 inhibitor) that manipulate p62 protein modifications can alter the ability of p62 to recognize and recruit autophagic cargo [164]. Additionally, targeting the activity of kinases involved in autophagy (e.g., ULK1 or TBK1, which modulate selective autophagy) could also provide benefits in protein quality control. However, these initiatives will require further studies in vivo and in vitro to determine how specific mutations alter autophagy and what potential avenues are available for therapeutic intervention.

**Table 1:** ALS-FTD genes and their disease mutations linked to autophagy

Gene symbol	Protein	Cellular functions	The effect of the genetic variants	Autophagy involvement
C9ORF72	C9orf72	Proteostasis and vesicle dynamics	Loss/gain of function	Regulates initiation of autophagy. [36, 37]
TBK1	TBK1 (TANK-binding kinase 1)	Proteostasis and immunity	Loss of function	Phosphorylates autophagy receptors (p62 and OPTN); regulates selective autophagy. [48–51]
OPTN	Optineurin	Proteostasis, vesicle trafficking and axon homeostasis	Loss of function	A substrate of TBK1 and autophagy receptor protein; selective autophagy. [25, 48, 49]
SQSTM1	p62	Pproteostasis, amino acid sensing, DNA damage response, and oxidative stress	Loss of function	A substrate of TBK1 and ULK1; autophagy receptor; selective autophagy.[25, 50, 68]
UBQLN2	Ubiquilin-2 (UBQLN2)	Proteasome, proteostasis, and vesicle trafficking	Gain/loss of function	A potential autophagy receptor. [88, 89]
TARDBP	TDP-43	RNA regulation	Loss/gain of function	Regulates autophagy initiation and autophagosome-lysosome fusion. [103]
FUS	FUS (fused in sarcoma)	RNA regulation, and DNA damage repair	Loss of function	ALS-linked mutations, P525L and R522G, impair autophagy. [165]
VCP	Valosin-containing protein	ER-associated degradation, DNA damage, and membrane dynamics	Loss of function	Regulates the clearance of lysosomes. [128]
SOD1	Superoxide dismutase 1	Dismutation reaction	Gain of function	Mutant SOD1 disrupts autophagy. [139–143]
ALS2	Alsin	Proteostasis and endosome biogenesis	Loss of function	Pathogenic mutations in ALS2 disrupt the formation of amphisomes. [150]
VAPB	Vesicle-associated membrane protein-associated protein B/C	Proteostasis, calcium homeostasis, and proteins trafficking	Loss of function	Regulates ER-mitochondrial contact. [155]
SigR1	Sigma receptor-1	Proteostasis, Ca^2+^ signaling, ion channel activity, synaptic plasticity.	Loss of function	Regulates autophagosome-lysosome fusion. [162, 163]

## Abbreviations

ALS: Amyotrophic lateral sclerosis
Atg: Autophagy-related genes
C9ORF72: Chromosome 9 open reading frame 72
CK2: Casein kinase 2
ER: Endoplasmic reticulum
FTD: Frontotemporal dementia
FTLD: Frontotemporal lobar degeneration
FUS: Fused in sarcoma
GEF: Guanine nucleotide exchange factor
GRD: Glycine-rich domain
HACE1: HECT domain and ankyrin repeat containing E3 Ub ligase 1
IBM: Inclusion body myopathy
IBs: Inclusion bodies
Keap1: Kelch-like ECH-associated protein 1
LC3: Microtubule-associated protein1 light chain 3
LIR: LC3 interacting regain
mTORC1: Mechanistic target of rapamycin complex 1
NBR1: Neighbor of BRCA1 gene 1
OPTN: Optineurin
PTPIP51: Protein tyrosine phosphatase interacting protein 51
RRM: RNA recognition motifs
SigR1: Sigma receptor-1
SMCR8: Smith-Magenis Chromosome Region gene 8
SOD1: Cu-Zn Superoxide Dismutase 1
TBK1: TANK-binding kinase 1
TDP-43: Transactive response DNA-binding protein 43
TFEB: Transcription factor EB
TRIM21: Tripartite motif-containing protein 21
UBA: ubiquitin-binding domain
UBQLN2: Ubiquilin 2
UBXD1: Ubiquitin Regulatory X (Ubx) Domain-Containing Protein 1
ULK1: Unc-51 Like Autophagy Activating Kinase 1
UPS: The ubiquitin-proteasome system
VAPB: Vesicle associated membrane protein associated protein B
VCP: Valosin containing protein
WDR41: WD repeat domain 41
WIPI2: WD-repeat protein interacting with phosphoinositides 2
YOD1: Yeast OTU deubiquinating enzyme 1
